# Physiological, patho-physiological, and potential therapeutic roles for neutrophils in cancer & beyond

**DOI:** 10.3389/fimmu.2026.1760161

**Published:** 2026-04-22

**Authors:** Connor McGarrity-Cottrell, Aoife McGinley, Natalia Becares, Jakub Lich, James Roper, Samuel Florence, Mihil Patel, Mark A. Exley

**Affiliations:** 1 Lift Biosciences, London Bioscience Innovation Centre, London, United Kingdom; 2 Department of Life Sciences, Imperial College, London, United Kingdom

**Keywords:** cancer, immune competence, immunotherapy, neutrophils, tumour microenvironment

## Abstract

Neutrophils are the most common type of white blood cell in the body, acting as crucial mediators of innate immunity. They initiate immune responses against invading microorganisms and threats like cancerous cells. Neutrophil infiltration is observed across inflammatory and autoimmune diseases and many cancers. Neutrophils orchestrate innate and adaptive immune responses to return the body to homeostasis, including after infections and preventing the development of tumours. While both protective and deleterious functions of neutrophils have been reported, this dual functionality reflects the heterogeneity of neutrophil subsets. Previously, neutrophils short lifespan resulted in their underappreciation. Recent advances in technology and methodology have drastically increased understanding of neutrophil biology, heterogeneity and plasticity, leading to a plethora of discoveries around the therapeutic potential of neutrophils, both through their direct cytotoxic effects to remove deleterious populations and through their ability to modulate other components of immunity. How to achieve this therapeutically with a rapidly-turned over population has proven elusive until now. This review highlights physiological and patho-physiological activities of neutrophil populations and their therapeutic potential and challenges. Recent and ongoing efforts to target or exploit neutrophil-type populations therapeutically have included engager-type antibody-based reagents and ‘off-the-shelf’ allogeneic cell therapy with and ‘Immuno-Modulatory Alpha Neutrophils’ (IMANs).

## 1 Introduction

Neutrophils, polymorphonuclear granulocytes of the innate immune system with reported phenotypic and functional plasticity, are the most abundant leukocytes in the circulatory system, making up 50–70% of peripheral leukocytes in humans (1, 2). Rough estimates suggest humans produce ∼1 billion neutrophils daily per kilogram of body weight at steady state, in the range of 10^11^ cells/day for a typical adult, and this may extend to 10 billion/Kg under inflammatory conditions (3).

Neutrophils are historically considered to constitute the first line of immune defence, protecting the host from pathogen assaults via multiple mechanisms, including degranulation, phagocytosis, ROS release and formation of neutrophil extracellular traps (NETs/NETosis) (4). Indeed, granulocyte transfusions can provide protection against severe bacterial and fungal infections (5, 6). The clinical benefits of granulocyte transfusions in cancer patients may include these antimicrobial properties, cancer patients often being immuno-suppressed by the tumour and also by treatments such as chemotherapy (5, 6). However, direct antitumour benefits have been harder to demonstrate conclusively (7).

In addition, neutrophils have homeostatic functions and regenerative activities (8). Emerging studies indicate that some neutrophils can present antigens, co-regulate T cell responses (9) and kill target cells in an antibody-dependent cellular cytotoxicity manner (ADCC), reviewed in (10).

Neutrophils are found at highest levels in bone marrow (BM), followed by spleen and lung, lower still in liver, intestine, muscle, skin, adipose and other tissues and lymph nodes (LNs), protecting against infections in all locations. Average murine neutrophil lifespan reportedly varies from just over a day in liver to ~3 days in BM, with other tissues in between. In humans, a lifespan of up to ~5 days has been reported, but contradicted by others claiming less than half a day (reviewed in 10).

## 2 Neutrophils in cancer: ‘good’ versus ‘bad players.

### 2.1 Overview – neutrophils in cancer – pro and anti-tumour roles, impacting therapies

Recent literature reviews have highlighted the increasingly recognized role of neutrophils in cancer progression, as well as limiting the efficacy of cancer therapies, through a variety of immunosuppressive and pro-tumorigenic mechanisms within the tumour microenvironment (TME) ​ (11, 12)​. Indeed, circulating Neutrophil/Lymphocyte ratio has become a recognised stratifier of patient risk, with elevated N/L correlating with poor prognosis in a range of cancers (13).

### 2.2 Neutrophil TME immunosuppression

**Figure 1:**
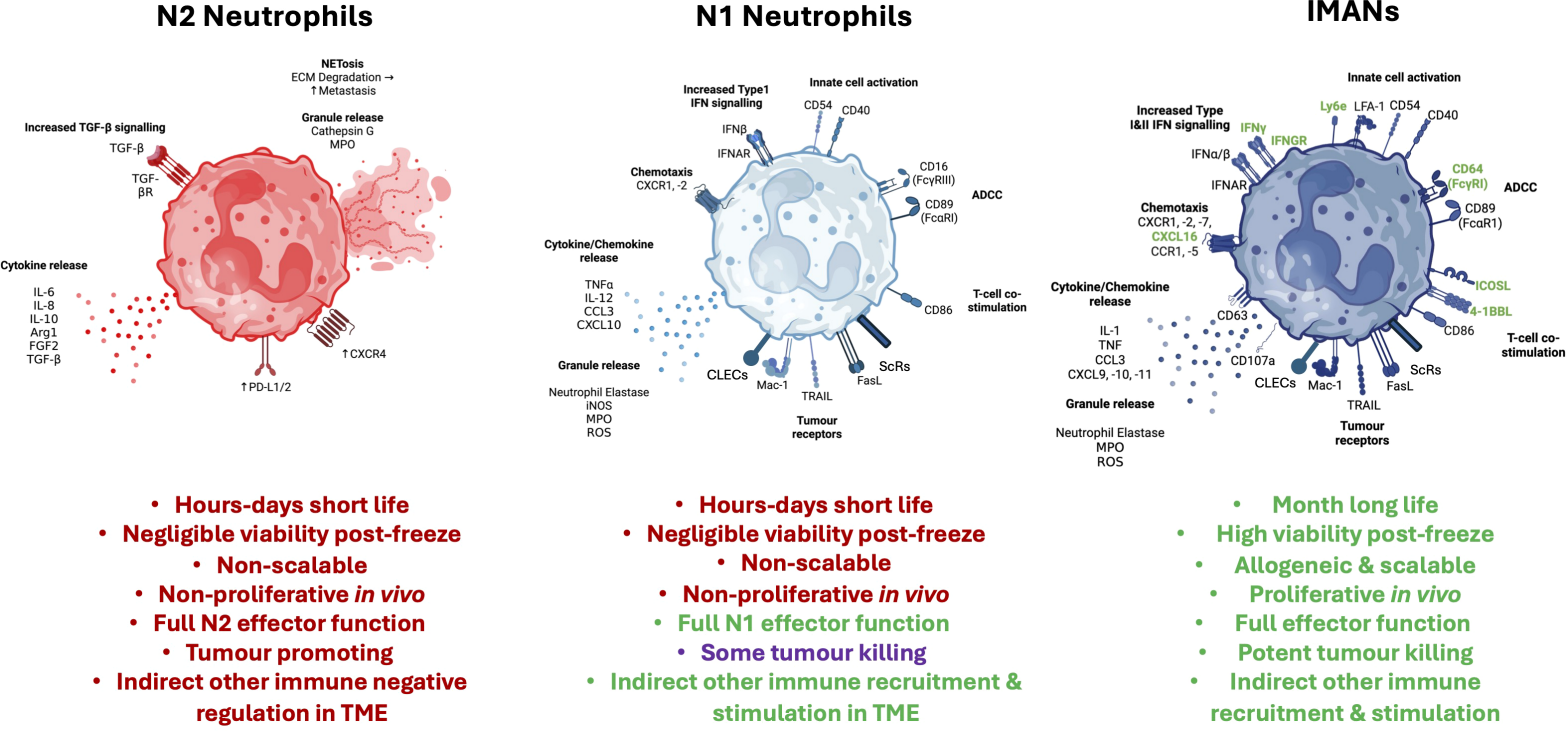
Main neutrophil types & IMAN™s. Main receptors, functional molecules and organelles of “N1” and “N2” neutrophils as extreme phenotype examples compared with IMANs for therapeutic potential with particular reference to anti-tumour responses. Colors are based on the general convention of red for inflammatory, blue for anti-inflammatory. IMANs color represents their optimized anti-tumor potential. N1 and N2 are only approximations of complex subsets, but practical functional distinctions. For the text, red is for ‘bad’ (in the context of anti-tumor responses, specifically) and green for ‘good’.

Once considered passive players in the tumour microenvironment, neutrophils are now understood to exert complex and often suppressive effects on anti-tumour immunity. Neutrophils are actively recruited into cancers and accumulate to high levels in many (14), notwithstanding their short lifespan, making this an underestimate of their total presence. Neutrophils, especially tumour-associated neutrophils (TANs) and polymorphonuclear myeloid-derived suppressor cells (PMN-MDSCs), can play paradoxical roles in cancer by supporting or hindering anti-tumour response and immunotherapeutic efficacy. In many particularly progressive tumours, the microenvironment induces the polarization of endogenous neutrophil precursors into relatively immunosuppressive ‘N2’-type phenotype (**Figure 1**) under the influence of cytokines such as TGF-β, IL-6, G-CSF, and GM-CSF ​ (15–17)​. These N2 TANs suppress antitumor immune responses by inhibiting T-cell activation, upregulating PD-L1 expression, and contributing to immune escape, all of which limit the effectiveness of immunotherapies, including checkpoint inhibitors ​ (18, 19).​ Moreover, TANs actively promote tumour progression through several mechanisms: they can release neutrophil extracellular traps (NETs) loaded with neutrophil elastase (NE) and matrix metalloproteinase-9 (MMP-9), which can remodel the extracellular matrix, awaken dormant tumour cells, and enhance metastatic spread (20, 21) (**Figure 1**). However, in patients that do better, anti-tumour ‘N1’-type TANs predominate (22–26) (**Figure 1**).

Further seminal work employing spatial and single-cell technologies have increasingly interrogated the composition of neutrophil subsets within the TME, suggesting that whilst the understanding of broad “N1” and “N2” pro- and anti-tumour phenotypes was groundbreaking, it may also be an oversimplification of the plasticity exhibited by patient neutrophils (27). This heterogeneity in neutrophil phenotypes becomes even more complex, not only across different cancer types, but also among patients within the same cohort who share similar disease biology. Moreover, marked differences in expression profiles of canonical neutrophil markers (e.g., CD66b^+^, CD11b^+^) observed between hepatocellular carcinoma and lung cancer (where each neutrophil subset is associated with distinct prognostic and disease severity outcomes) underscore the need for detailed characterisation of these populations to support the development of endogenous neutrophil targeting/modulating therapies to improve clinical outcomes (27–29).

### 2.3 Tumour metastasis, resistance & neutrophils

Another pro-tumourigenic mechanism elicited by TANs is driving epithelial-mesenchymal transition (EMT) via pathways such as JNK-ZEB1/Snail, facilitating tumour cell invasion and lymph node metastasis. In addition, TANs contribute to angiogenesis by secreting VEGF, Bv8, FGF2, and other proangiogenic molecules, thus supporting tumour growth and enabling resistance to anti-angiogenic therapies (11).

These pro-tumour activities are compounded by their ability to create a suppressive tumour microenvironment that fosters therapeutic resistance and tumour relapse. As a result, high TAN infiltration is often associated with poor prognosis and treatment failure in many cancers, including colorectal, lung, and gastric cancers (11, 12). Part of this treatment resistance is believed to be promoted by NETosis of infiltrating TANs ​ (30, 31)​. Recently, Mousset A et al. showed that two NET-associated proteins are central to this process: integrin αvβ_1_ (ITGαvβ1), which sequesters latent TGFβ, and matrix metalloproteinase 9 (MMP9), responsible for cleaving and activating trapped TGFβ ​ (30)​. Importantly, in their study the authors showed that blocking any component of this IL1β–NET–TGFβ axis reinstates chemotherapy efficacy. Therefore, inhibitors that target NET formation (via PAD4 or DNase I), IL1β, MMP9, TGFβ signaling, or ITGαvβ1 can potentially significantly reduce metastasis, reverse EMT, and enhance the therapeutic response both *in vitro* and *in vivo*.

Emerging strategies in cancer therapy now aim to counteract these effects by reprogramming TANs toward an antitumour phenotype, inhibiting their recruitment, or targeting their tumour-promoting signaling pathways, offering a promising adjunct to existing treatments. Consequently, therapeutic strategies aimed at reprogramming or depleting pro-tumourigenic neutrophil subsets are gaining attention as potential adjuncts to standard cancer treatments. Collectively, these findings underscore the need for strategies that target neutrophil recruitment (e.g., CXCR2 inhibitors) or reprogram their function to enhance the efficacy of existing cancer treatments.

## 3 Neutrophils enhancing existing cancer therapies

### 3.1 Neutrophils and adoptive T-cell therapies (CAR-T + TCR-T)

Chimeric antigen receptor (CAR) T cells are autologous T lymphocytes engineered with synthetic receptors that redirect specificity toward surface antigens restricted to, or upregulated on tumour cells. This enables cytotoxicity based on surface recognition in contrast to physiological T-cell activation which is based on peptide-MHC recognition. Lysis of target cells occurs following target engagement and signalling through the intracellular signalling domains. To date, clinically approved CAR-T therapies are used in haematological cancer and target CD19 or B-cell maturation antigen (BCMA). It is within these indications where most of the clinical success has been observed. Impressive complete remission has been observed in B-cell leukaemia, lymphoma and multiple myeloma (32–34). Success in solid tumours has been limited due to the absence of truly tumour specific antigens and the risk posed by on target off tumour activity (35).

Given that neutrophils are the most common cell type in the blood, their predominance in solid tumours, and their effect on T-cells immunotherapies highlighted above it is reasonable to consider that these cells influence the persistence and efficacy of CAR-T or even TCR-T cells. One of the most common complications of CAR-T therapy is Cytokine Response Syndrome (CRS), which occurs in most patients (36). Humanised mouse studies showed that CRS is driven by monocytes and macrophages releasing IL-1 and IL-6 (37, 38). Whilst these cells are the major source of the cytokines, this release was shown to be preceded by a spike in neutrophil activation (39). In this study 26 patients with multiple myeloma who were treated with ciltacabtagene autoleucel (anti-BCMA CAR-T cells) were monitored longitudinally to assess their cytokine profile, immune cell phenotype and gene transcriptome. Gene set enrichment analysis showed that neutrophils responded as early as day 3-5, whilst the other immune cells were activated later at the onset of fever suggesting that neutrophils may initiate the inflammatory cascade. This is in line with the pathophysiology of COVID19, where NETs were found to be a contributor to virus induced lung damage (40). A similar study which also looked at 26 patients longitudinally, but following CD19 CAR-T treatment, looked at 453 protein markers in the blood. This study showed CD33, Myeloperoxidase (MPO) and DEF1a (all expressed by neutrophils) to be increased in the lead up to, or at the onset of peak CRS (41). CRS has been reported to be correlated with more favourable clinical outcomes, therefore the recent findings showing upregulation of neutrophil markers as a harbinger of CRS may point to neutrophils, or a subset thereof, being positively associated (42).

In one prospective study elevated immature (CD10^-^) neutrophils was found to correlate with poorer prognosis, worse treatment response and reduced CAR-T persistence (43). These cells had been previously found in Non-Hodgkins Lymphoma patients and exhibited MDSC like biology, such as inhibiting T-cell proliferation and having an immature phenotype. One neutrophil relevant biomarker that that can be correlated with outcomes in response to immunotherapeutic interventions is the neutrophil-to-lymphocyte ratio (NLR), which is calculated as a ratio of neutrophil and lymphocyte counts measured in peripheral blood. A higher NLR has been significantly associated with poorer outcomes in a range of inflammatory conditions such as heart disease, influenza and chronic kidney disease (and for that matter all cause mortality in the general population) (44). In relation to solid tumours, one study found that elevated NLR was significantly associated with lower Overall Response Rate (ORR; 4.2% vs. 55.9%, P <0.001) and Overall Survival (OS; 5.6 vs. 13.8 months, P< 0.001). Single cell RNA sequencing of blood from anti-Claudin 18.2 CAR-T patients revealed a district subset of neutrophil at increased levels in disease progression and which had elevated MMP9 and IL-17 in the STING signalling pathway (45).

Thus, rather than concluding whether neutrophils may have positive or negative effects on CAR-T treatment response, it would be of far greater prognostic value to take into account the plasticity and heterogeneity of neutrophils and uncover more specific biomarkers within subsets of neutrophils (46).

### 3.2 Neutrophils and immune checkpoint inhibitors/antibody-based therapies

#### 3.2.1 Neutrophils enhancing mAb activity

Until relatively recently, neutrophils have been largely overlooked when considering Fc receptor-mediated tumour killing, but are increasingly recognised as critical effector cells for therapeutic antibodies, functioning as abundant Fc receptor-expressing leukocytes that can substantially enhance anti-tumour efficacy. In addition to their canonical role in host defence, neutrophils express a broad panel of Fcγ receptors, including FcγRIIa and FcγRIIIb constitutively and, upon cytokine priming, high-affinity FcγRI, enabling them to respond efficiently to IgG1 monoclonal antibodies used in cancer therapy. Following opsonisation of malignant cells, cross-linking of these Fcγ receptors triggers a multifaceted cytotoxic programme encompassing Antibody-Dependent Cellular Cytotoxicity (ADCC) through a Reactive Oxygen Species (ROS)-mediated respiratory burst, degranulation, and the engagement of death ligands such as FasL and TRAIL that induce caspase-dependent apoptosis of tumour cells (47).

Neutrophils also mediate Antibody-Dependent Cellular Phagocytosis (ADCP) of smaller targets and a distinctive trogocytic process against larger tumour cells, in which repeated extraction of antibody-opsonised membrane fragments ultimately results in loss of plasma membrane integrity and necrotic cell death. Under conditions of strong Fc receptor engagement, neutrophils can further release neutrophil extracellular traps (NETs) composed of DNA and histones decorated with granule proteins, which can immobilise and damage antibody-coated tumour cells. Consistent with these mechanisms, depletion of neutrophils in preclinical models can attenuate the anti-tumour activity of clinically relevant antibodies, demonstrating that neutrophils are required for therapeutic efficacy alongside more understood effector cells, such as macrophages and natural killer (NK) cells in an anti-cancer context (48, 49).

The importance of neutrophils becomes evident when antibody formats are tailored to their Fc receptor repertoire. IgA monoclonal antibodies and CD89 (FcαRI)-targeting constructs have emerged as powerful tools to recruit neutrophils as dominant effector cells. FcαRI is constitutively expressed on human neutrophils and signals via associated FcRγ chains; because each FcαRI–FcRγ complex can engage two IgA Fc regions and activate multiple ITAM motifs, IgA antibodies can induce more potent Fc receptor signalling than IgG, when tested molecule for molecule (48, 50). Quantitative signalling studies demonstrate enhanced Syk phosphorylation, ERK activation, and reactive oxygen species generation downstream of FcαRI engagement, with receptor clustering thresholds lower than those required for FcγR activation (50, 51). Consistent with this signalling cascade, tumour-directed IgA antibodies elicit stronger intracellular activation and superior neutrophil-mediated killing of cancer cells compared with matched IgG antibodies across multiple antigen systems, including EGFR, HER2, and CD20 (52–54).

Functionally, these signalling differences translate into measurable potency advantages. *In vitro*, IgA antibodies typically demonstrate 5–20-fold lower EC50 values for neutrophil-mediated ADCC and achieve maximal tumour cell lysis of 60–90% in contrast to 20–40% lysis for IgG under comparable effector-to-target ratios (52, 53). Enhanced cytotoxicity reflects amplified trogocytosis, oxidative burst, and antibody-dependent phagocytosis, all of which scale with FcαRI clustering efficiency (51). In human FcαRI transgenic xenograft models, these functional gains translate into significantly improved tumour control, with prolonged tumour growth delay, higher complete response rates, and neutrophil-dependent survival benefits compared with matched IgG antibodies (52, 54). Mechanistically, IgG efficacy in neutrophils is partially constrained by the high expression of the GPI-anchored FcγRIIIb, which can sequester IgG immune complexes away from signalling-competent Fcγ receptors, whereas FcαRI engagement is not subject to equivalent disruption (48, 52).

Despite these potency advantages, translational performance is strongly influenced by differences in the pharmacokinetic profiles between isotypes. IgA exhibits a substantially shorter serum half-life (approximately 3–6 days) than IgG (~21 days), limited FcRn recycling, and enhanced clearance, resulting in reduced systemic exposure despite higher per-engagement effector activity (52, 53, 55). Consequently, therapeutic impact depends not only on intrinsic cytotoxic potency, but also on exposure kinetics and tumour microenvironment composition. Seminal studies and dominant themes within the literature currently suggest that IgA superiority is most pronounced in neutrophil-rich tumours, whereas IgG may retain advantages in macrophage-dominant environments or where prolonged systemic exposure is required (53–56). These findings support the concept that isotype switching of tumour-specific monoclonal antibodies from IgG to IgA can substantially enhance neutrophil-mediated antitumour activity, but that optimal clinical efficacy will likely depend on pharmacokinetic optimisation and tumour microenvironment (TME) context (52–56).

Beyond monovalent Fc-receptor interactions, bispecific antibody formats provide an opportunity to engage neutrophils and other myeloid cells more selectively. Li et al. designed a bispecific antibody that co-targets CD20 on malignant B cells and human CD89, enabling direct recruitment of CD89-expressing myeloid effectors to CD20^+^ tumour cells (57). *In vitro*, this CD20×CD89 bispecific antibody induced robust antibody-dependent cytotoxicity and phagocytosis by monocytes and macrophages, and in human CD89-transgenic mouse models it exhibited pronounced anti-tumour efficacy, including in settings where conventional anti-CD20 IgG antibodies were less effective (57). Although the emphasis in that study was on macrophage-mediated effects, the same principle is readily applicable to neutrophils, which express high levels of CD89 and can be efficiently recruited by CD89-directed bispecifics (48). By physically bridging a tumour-associated antigen with CD89, bispecifics can concentrate neutrophils at the tumour cell surface, promote stable adhesion via integrins such as aMb2, and trigger potent ADCC, trogocytosis and degranulation. More broadly, CD89-targeting bispecific and Mult specific constructs exemplify a wider therapeutic strategy in which the abundant circulating pool of neutrophils is deliberately mobilised against tumour cells, and tumour-associated macrophages (particularly immunosuppressive “M2-like” subsets), are re-educated into active cytotoxic effectors (48, 57).

Neutrophil-based potentiation of antibody therapy extends beyond direct cytotoxicity at the tumour cell interface. Firstly, neutrophil function and Fc receptor expression are highly dynamic and modifiable with cytokines. Exposure to GCSF, GM-CSF, type I and type II interferons prolongs neutrophil survival, enhances their respiratory burst capacity, and up-regulates high-affinity FcγRI expression, thereby lowering the threshold for activation by IgG1 therapeutic antibodies and acting as a priming mechanism (48, 49). Such cytokines can be co-administered or incorporated into antibody fusion proteins to locally prime neutrophils within the TME. Secondly, neutrophils express ligands of the TNF superfamily, including FasL and TRAIL, and are amenable to arming with additional death-inducing ligands via fusion proteins or bispecific designs; for example, preclinical work has shown that targeting TRAIL to neutrophils can introduce a parallel death receptor-mediated apoptotic pathway that acts in concert with Fc receptor-driven ADCC against tumour cells (48, 49). Thirdly, by phagocytosing or trogocytosing antibody-opsonised tumour cells, neutrophils generate antigen-rich cellular debris and release inflammatory mediators that can be captured by professional antigen-presenting cells, thereby facilitating cross-presentation and the priming of tumour-specific CD8^+^ killer T-cell responses (48, 49). In this way, neutrophil engagement by tumour-targeting antibodies not only enhances immediate tumour cell killing but may also contribute to the development of durable adaptive anti-tumour immunity.

Collectively, the growing body of evidence positions neutrophils as versatile, programmable Fc receptor-expressing effector cells that can be exploited to augment the efficacy of antibody-based cancer therapies. Conventional IgG1 monoclonal antibodies already recruit neutrophils via FcγR-dependent ADCC, ADCP, trogocytosis and NET formation, and neutrophil depletion consistently diminishes their anti-tumour activity *in vivo* (48, 49). Logical manipulation of Fc–receptor interactions through isotype selection (e.g. IgA), Fc engineering and CD89-engaging bispecific antibodies can shift a greater proportion of effector function onto neutrophils and other myeloid cells, leading to more robust tumour clearance (48, 52, 57). When combined with cytokine priming and death ligand-based fusion constructs, these strategies offer pathways by which neutrophils are integrated into next-generation antibody therapies, with the potential not only to increase direct cytotoxicity but also to reshape the TME and support effective longer lasting T cell-mediated immunity.

#### 3.2.2 Neutrophils enhancing immune checkpoint inhibitor therapies

Neutrophils are now recognised as major determinants of response and resistance to immune checkpoint inhibitor (ICI) treatment. In many solid tumours, tumour-associated neutrophils (TANs) and an elevated peripheral neutrophil to lymphocyte ratio (NLR) correlated with poor prognosis and reduced benefit from PD-1/PD-L1 or CTLA-4 blockade. A large meta-analysis across >4000 ICI-treated patients showed that rising NLR early on therapy is consistently associated with shorter overall and progression-free survival and lower response rates, whereas a falling ratio is associated with clinical benefit (58). Post-treatment, a high NLR appears even more prognostic than baseline values, emphasising that neutrophil dynamics under ICI pressure reflect evolving resistance. Similarly, high NLR has been linked to hyper-progressive disease after ICIs, suggesting that neutrophil-skewed myelopoiesis can predispose to paradoxical tumour acceleration (59).

Mechanistically, TANs are known to shape several key features of ICI resistance. Faget et al. outline four interconnected axes where TANs can favour a pro-tumour environment: (1) adaptive immune suppression, where TANs (and PMN-MDSCs) inhibit CD8^+^ T-cell proliferation and cytokine production, and recruit Tregs to create a persistent immunosuppressive environment (2) neo-angiogenesis, assisted by neutrophil-derived VEGF, which may also underlie resistance to anti-VEGF plus ICI combinations; (3) immune exclusion, as dense neutrophil infiltrates at the invasive front correlate with poor T-cell and macrophage entry into tumour nests; and (4) interaction with tumour-intrinsic pathways, where oncogenic signalling (e.g., EGFR, MET, PI3K or Wnt/β-catenin) favours recruitment of tumour-promoting neutrophils and reduced ICI sensitivity (60). In Non-Small Cell Lung Cancer (NSCLC), for example, TANs are the dominant leukocyte population (~20% of CD45^+^ cells), their abundance inversely correlates with CD4^+^ and CD8^+^ T-cell content, and they support proliferation, metastatic spread and stemness via ROS, proteases and pro-growth factors (61). These observations align with broader neutrophil biology, where pro-tumour N2/Polymorphonuclear Myeloid-Derived Suppressor Cells (PMN-MDSC)-like states and high systemic NLR are repeatedly associated with attenuated ICI benefit.

Together, these studies suggest neutrophils are both biomarkers and active modifiers of checkpoint inhibitor efficacy, often with a negative association with disease progression. However, the complexities of the many varying immune cells within a dynamic TME are perhaps not captured within these studies as increasingly we are understanding that particular immune subsets can markedly influence the overall response to treatment, both positively and negatively.

Neutrophils are highly plastic with high functional diversity, and specific subsets can enhance checkpoint blockade. Preclinical work demonstrates that type I/II interferons and TGF-β blockade can skew TANs toward an N1-like, anti-tumour state with increased cytotoxicity and support for CD8^+^ T-cell responses. Benguigui et al. recently identified interferon-stimulated Ly6E^hi^ neutrophils as a blood-borne biomarker and functional mediator of anti-PD-1 response: these cells are induced by tumour-intrinsic STING–IFN signalling, directly activate cytotoxic T cells via IL-12b, and their frequency at baseline predicts ICI outcome across mouse models and human melanoma/NSCLC cohorts with high accuracy (26). Further *in vivo* animal work has shown that STING pathway activation following the induction of tumour senescence promoted the infiltration of neutrophils with a concomitant activation of cytotoxic T cells, and this anti-tumour activity was enhanced in the presence of ICIs (62).

These studies confirm it is the balance between neutrophil subsets, rather than bulk counts, that determine whether these cells undermine or potentiate checkpoint blockade, and that re-programming or replacing TANs represents a rational strategy to potentially improve ICI efficacy (63). So far, approaches that aim to eliminate or inactivate pathological pro-tumour MDSCs have revealed their challenges and point to the need for more effective targeting of all types of immunosuppressive cells that contribute to the pro-tumour TME, or indeed therapies that tip the balance in favour of the patients own immune defenses.

## 4 Direct neutrophil therapeutic potential

Owing to their ability to both modulate immune responses and act as effector cells, multiple strategies target endogenous neutrophils or aim to replace the defective ones in patients. ‘Off-the-shelf’ cell therapies are feasible in refractory cancer patients. Indeed, decades of widespread clinical whole blood and granulocyte transfusions demonstrate the safety of allogeneic neutrophils from many donors (5, 6). The clinical benefits of granulocyte transfusions have been reported in trials in antimicrobial resistance, though more inconclusive in cancer patients, other than for these antimicrobial properties (5–7).

Recently, three Haemopoietic Stem Cell (HSC)-derived allogeneic stem cell-based products with major myeloid components have gained regulatory approval in multiple countries through successful multi-stage clinical trials. As these cells mature *in vivo*, a significant proportion of each become neutrophil-like cells. Romyelocel-L (CLT-008), an allogeneic myeloid progenitor cell therapy, demonstrated a favourable safety profile, transient *in vivo* persistence, and no induction of graft-versus-host disease in clinical trial in oncology patients (64). Romyelocel-L decreased incidence of infections, antimicrobial use, and hospitalization in Acute Myeloid Leukemia patients receiving chemotherapy, immunosuppressed by both disease and treatment (64). Omisirge^®^ (Omidubicel) as a source of stem cells for HSC Transplant (HSCT), resulted in faster neutrophil, platelet, B cell, T cell, NK cell and monocytic lineage recovery, as well as reduced infections compared with umbilical cord blood transplant in haematologic malignancies or sickle cell disease, along with high and durable engraftment rates (65–69). Zemcelpro^®^ (UM171-expanded CD34^+^ cells; Dorocubicel; 69) was recently granted conditional marketing authorisation in the European Union for adults undergoing allogeneic haematopoietic stem cell transplant, based on successful clinical trials, providing contemporary regulatory precedent for the clinical use and oversight of UM171-expanded progenitor-based allogeneic cell therapy. Zemcelpro^®^ is also an engrafting HSCT-type therapy resulting in rapid neutrophil, platelet and T cell reconstitution (69).

Immuno-modulatory alpha neutrophils (IMANs^®^) are a neutrophil-related allogeneic long-lived and cryopreservable treatment produced from haemopoietic stem cells (70). IMANs were designed for optimal potential anti-tumour activity and both kill tumour cells and recruit and activate T cells and NK cells into tumours *in vivo* and *in vitro* (70). Optimal ‘N1’ anti-tumour neutrophil phenotypes were described as dominant in patients that do best spontaneously as well as with immunotherapy, compared to ‘N2’ dominance in patients that progressed, as well as similar results multiple *in vivo* models (22–26) (**Figure 1**). IMANs have such N1 characteristics fixed into their biology, with the additional benefits of lasting about a month in this anti-tumour state and scalable ‘off-the-shelf’ capability, making them therapeutically relevant (70) (**Figure 1**). IMANs also have potential in other diseases (71), again based on independent findings in those areas of the importance of the right populations of neutrophils and defining those phenotypes versus the negative impact of incorrectly polarised patient neutrophils, where disease progresses (72–77; Tang et al., 22; 78, 79). These findings arise from early-stage preclinical studies (encompassing both *in vivo* and *in vitro* models), with additional experimental validation in progress to further strengthen the dataset ahead of formal scientific publication (70, 71).

## 5 Targeting neutrophils in other therapeutic areas – AMR, neuro, autoimmunity, longevity

Roles for neutrophils in many disease areas are now known, several being found to have therapeutic implications in recent studies, as briefly summarized below.

### 5.1 Antimicrobial resistance

Antimicrobial resistance (AMR) is escalating worldwide, driven by overuse of antibiotics, limited new drug development, and bacterial evolution (80). Neutrophils and therapies that recruit or enhance their activity have shown promising potential against resistant infections. Payne et al. developed a novel antibiotic-chemoattractant linking the antibiotic vancomycin to a neutrophil-recruiting peptide (fMLF), which both killed *Staphylococcus aureus* through enhanced neutrophil chemotaxis, phagocytosis, and bacterial clearance *in vitro* and in an *in vivo* MRSA model, achieving up to five-fold lower antibiotic doses than vancomycin alone (76). Similarly, Gao et al. used neutrophils as living carriers to deliver colistin and azithromycin to infection sites, reducing bacterial loads in mouse lungs by over 2 logs and improving survival from 40% to 90% compared with conventional antibiotics (77). These studies highlight the potential of therapies that harness neutrophils to enhance bacterial clearance and tackle AMR.

### 5.2 Neurodegenerative diseases, autoimmunity & inflammation

Neutrophils are increasingly recognised as key drivers of neurodegeneration, autoimmunity, and chronic inflammation; where their proteases, reactive oxygen species, cytokines, and NETs can both damage and repair tissue. Modulating neutrophil activity or targeting specific subsets is emerging as a promising therapeutic strategy in conditions such as multiple sclerosis, rheumatoid arthritis, and Alzheimer’s disease. Zhou et al. identified CD177^+^ neutrophils as a functionally activated subset that negatively regulates inflammatory bowel disease (IBD). These neutrophils were shown to produce anti-inflammatory cytokines including IL-10 and TGF-β, while limiting pro-inflammatory mediators such as TNF-α and IL-6, thereby reducing intestinal inflammation. In both patient samples and mouse models, higher frequencies of CD177^+^ neutrophils correlated with reduced disease severity, improved histopathology, and decreased leukocyte infiltration; whereas depletion of this subset exacerbated colitis (73). The study highlights that neutrophil heterogeneity is a key determinant of inflammatory outcomes and suggests that selectively enhancing CD177^+^ neutrophils could represent a novel therapeutic strategy in autoimmune and inflammatory diseases. Further commentary by Salas et al., emphasises that certain neutrophil populations in ulcerative colitis can promote mucosal healing, clear microbial threats, and restore intestinal homeostasis, highlighting that selectively enhancing beneficial neutrophils may offer a novel therapeutic strategy in autoimmune and inflammatory diseases (78).

#### 5.2.1 Neurology

Neutrophils, traditionally seen as short-lived inflammatory responders, can also support CNS repair by delivering early repair signals and shaping a regenerative microenvironment. Kurimoto et al. demonstrated that neutrophils are a key source of the growth factor Oncomodulin (Ocm), essential for optic-nerve regeneration in mice. Following intraocular zymosan-induced inflammation, >90% of Gr-1+ neutrophils expressed Ocm within 6–12 h. Neutrophil depletion markedly reduced retinal Ocm and significantly suppressed axon regeneration (p < 0.05–0.001). Exogenous Ocm nearly doubled neurite outgrowth *in vitro*, whereas an Ocm antagonist peptide reduced regeneration to levels seen with neutrophil depletion (p < 0.001). This, together with the finding that macrophages could not compensate, underscores the early and indispensable role of neutrophils in promoting CNS axon regeneration (72). Furthermore, Sas et al. identified a distinct subset of neutrophils that promote CNS neuron survival and axon regeneration. In mouse optic-nerve and spinal-cord injury models, neutrophils recruited 3 days post-injury secrete factors such as NGF and IGF-1, significantly enhancing neurite outgrowth *in vitro* and axon regeneration *in vivo*, whereas early infiltrating or bone-marrow neutrophils did not. Neutralization of NGF and IGF-1 abrogated this effect, highlighting the critical role of neutrophil-derived growth factors in driving neuro-regeneration (75).

#### 5.2.2 Implications of neutrophil therapeutic potential: inflammaging & longevity?

In addition to all the functions previously discussed within this review, neutrophils are increasingly recognised for their roles in maintaining tissue homeostasis and influencing aging processes. Beyond their immune defense roles, they can modulate “inflammaging” (age-related immune decline) and support longevity by clearing senescent or damaged cells and promoting tissue remodeling. Binet et al. displayed that neutrophils actively target senescent retinal vasculature via neutrophil extracellular traps (NETs) decorated with granule proteins such as neutrophil elastase. This NET-mediated clearance of dysfunctional endothelial cells promoted regenerative revascularization and improved retinal structure and function. Depletion of neutrophils or inhibition of NET formation impaired vascular remodeling, leading to persistent pathological angiogenesis. These findings highlight a beneficial role for neutrophils in eliminating senescent cells and maintaining tissue health, suggesting that harnessing neutrophil-mediated clearance could mitigate age-related vascular decline and promote longevity (74). Similarly, neutrophils may also exert beneficial effects in tumour contexts by clearing senescent cells. Favaretto et al. demonstrated that Palbociclib-induced senescent breast cancer cells release a neutrophil-activating secretome, including IL-8 and SAA1, which recruits and activates neutrophils. These neutrophils respond by forming NETs, generating reactive oxygen species, and efficiently phagocytosing senescent tumour-cell debris, suggesting that neutrophil-mediated clearance of senescent or damaged cells can remodel the tumour microenvironment and potentially restrain tumour progression (79). Together, these findings underscore the emerging concept that harnessing neutrophil-mediated clearance of senescent or damaged cells may represent a promising therapeutic strategy to support tissue health, counteract age-related immune decline, and potentially modulate tumour progression.

Multiple other approaches target neutrophils. One established target is myristoylated alanine-rich C kinase substrate (MARCKS) protein, for which inhibitors such as BIO-11006 and Myristoylated Alanine-Rich C Kinase Substrate N-terminal Sequence (‘MANS’) have been designed and which function *in vitro* and *in vivo* (81). These inhibitors can suppress neutrophil inflammation (81, 82). This can be via β2-integrin activation and signaling (83). However, part of their function can be through monocytes as well (84).

## 6 Conclusions & prospects for neutrophil-directed therapies

Clinicians have always concentrated on neutrophils, one of the most immediate and regularly measured of clinical parameters, due to their critical importance in health and disease. However, in research, neutrophils have been relatively ‘forgotten cells’ of the immune system, ‘hiding in plain sight’ due to their short lifespan and common experimental focus on other easier to work with (mostly mononuclear) immune cells. The last few years have seen a renaissance in understanding of neutrophil biology as well as an acknowledgement of the multiple subsets, individual biology and difficulty of working with these definitively (3, 4, 11, 12). Neutrophil populations have been found to either positively or negatively regulate immunity, homeostatic and repair mechanisms, which has raised interesting questions about the potential of neutrophil-based therapies across a broad spectrum of diseases beyond traditionally considered anti-microbial/anti-bacterial roles (8–11). These areas include a major focus on cancer, but also include neurodegenerative diseases, autoimmunity and inflammation, potentially ‘as far as’ longevity. Neutrophil-based therapies appear to be a feasible goal in the next few years and safety appears likely based upon long experience with blood and granulocyte transfusions, as well as recent approvals of Haemopoietic Stem Cell-based therapies that produce neutrophils among other cell types after administration *in vivo*. As neutrophil-related clinical trials are planned, we see fundamental questions begin to be addressed with important takeaways that will inform future cell therapy studies:

1. How do we best direct neutrophil immunosurveillance to durable remissions and possibly cures.
2. What is the feasibility of **‘**off-the-shelf**’** products, what should be their ideal capabilities (e.g. optimal neutrophil subsets for each indication)?
3. What are rational combinations of optimized neutrophil-directed and other therapies to co-administer?

While future clinical trials are anticipated to address these points, **‘**innate immune**’** neutrophil populations can exert both effector and immunomodulatory functions, and may not require chemotherapy preconditioning (unlike **‘**niche-seeking**’** HSCT, CAR-T cells etc.), substantially expanding their therapeutic reach.

With these strengths and building on original blood and granulocyte transfusions and more recent neutrophil-related HSC-derived cellular immunotherapies, optimized neutrophil-based therapies have the potential, alone and in combination, to positively impact many patients in the future.
